# A comparative study of the debridement efficacy and apical extrusion of dynamic and passive root canal irrigation systems

**DOI:** 10.1186/1472-6831-14-12

**Published:** 2014-02-11

**Authors:** Ahmed Alkahtani, Tala D Al Khudhairi, Sukumaran Anil

**Affiliations:** 1 Department of Restorative Dental Sciences, College of Dentistry, King Saud University, Riyadh, Kingdom of Saudi Arabia; 2 Department of Periodontics and Community Dentistry, College of Dentistry, King Saud University, Riyadh, Kingdom of Saudi Arabia

**Keywords:** EndoVac, Extrusion, Irrigation, Side vented needle, Tip vented needle

## Abstract

**Background:**

Root canal irrigation carries a risk of extrusion of irrigant into the periapical tissues which can be associated with pain, swelling, and tissue damage. Studies have shown less extrusion with sonic or apical negative pressure devices compared with syringe and side-port needle or passive ultrasonic irrigation with continuous irrigant flow. This study aimed to evaluate the effectiveness of the EndoVac irrigation system, regarding 1) debris removal and 2) the control of apically extruded irrigating solution.

**Methods:**

Fifty extracted human single-rooted teeth were used in this study. The teeth were then randomly divided into three experimental groups according to the type of irrigation used and one control group. In group 1, irrigation was performed using the EndoVac irrigation system. In group 2, irrigation was performed using a 30-gauge, tip-vented irrigation needle. In group 3, irrigation was performed using a 30-gauge, side-vented irrigation needle. The control group received instrumentation with no irrigation to serve as a control for cleaning efficiency. Root canal instrumentation was performed using the Profile NiTi rotary system with a crown-down technique. All of the experimental teeth were irrigated with the same amount of 5.25% sodium hypochlorite. The amount of extruded irrigating solution was then measured by subtracting the post-instrumentation weight from the pre-instrumentation weight using an electronic balance. The cleanliness of debris removal was evaluated using scanning electron microscopy.

**Results:**

EndoVac irrigation had the least amount of extrusion followed by the side-vented and tip-vented method. The difference between the groups was statistically significant (P <0.01). As for the cleaning results, the debris collection in the EndoVac and tip-vented groups was the least in the apical third. In the control and the side-vented groups, the debris was the greatest in the apical third, but this difference was not significant among the three experimental groups.

**Conclusions:**

The EndoVac irrigation system extruded significantly less irrigant solution than either needle irrigation system. Debris collection was the least in the apical third for the EndoVac irrigation system. No significant difference was found in the cleaning efficiency among the three irrigation systems.

## Background

Successful endodontics is based on the sound principles of debridement, disinfection, and obturation aimed at maintaining dentition and providing an environment conducive to periradicular healing. Instrumentation, disinfection and obturation are important aspects of quality endodontic care. The eradication of the vital and necrotic remnants of pulp tissue and its debris, microorganisms and microbial toxins from the root canal system is the main goal of instrumentation [1]. Cleaning and debriding the root canal system involves the removal of organic and inorganic debris. The organic debris includes the vital and necrotic pulp tissue, microorganisms, salivary or tissue fluids, endotoxins and other foreign components that have entered the root canal system. In contrast, the inorganic debris includes the minerals that are deposited in the canal system and debris deposited on the canal walls subsequent to instrumentation [2,3]. Root canal irrigation is the key to cleaning and disinfecting the areas where the instrument cannot reach [4]. Disinfection is composed of the removal of the residual tissue in the canal system and the associated bacteria by flushing the canal system with irrigating solution. The objective is to remove as much residual tissue as possible [5].

Several studies have demonstrated that proportionally large areas of the main root canal wall remain untouched by instruments, especially in the apical third [3,6,7]. There is no single irrigating solution that fulfils the requirements of an ideal irrigant. The optimal irrigation is based on the combined use of two or more irrigating solutions using a proper irrigating technique [3]. Irrigants have traditionally been delivered to the root canal space with positive pressure and needles of different sizes and tip designs. The most apical part of the main root canal is where the irrigant should ideally be delivered [8]. Therefore, many of the irrigants have been chemically modified, and several mechanical devices have been developed to improve the penetration and effectiveness of irrigation. The apical preparation size also affects irrigant replacement in the root canals [9,10].

The EndoVac (Discus Dental, Culver City, CA) irrigation system, with its apical negative pressure, is able to thoroughly remove the micro debris at the apical constricture, thereby providing a better environment to be filled with sealer [1,11], without forcing the solution out of the apex into the periapical tissue. The system utilises apical negative pressure through the high-volume evacuation system, permitting a thorough irrigation with a high volume of irrigation solution [12,13]. Nielsen and Craig Baumgartner [14] found that the volume of irrigant delivered with the EndoVac system was significantly more than the volume delivered with needle irrigation over the same amount of time. Furthermore, they reported significantly better debridement for the EndoVac system compared to needle irrigation.

Root canal irrigation carries a risk of extrusion of irrigant into the periapical tissues which can be associated with pain, swelling, and tissue damage [15,16]. Studies have shown less extrusion with sonic or apical negative pressure devices compared with syringe and side-port needle or passive ultrasonic irrigation with continuous irrigant flow [3,17].

This *in vitro* study was aimed to evaluate the efficacy of apical cleaning and the extent of extruded irrigating solution using the EndoVac irrigation system compared to the tip-vented and side-vented root canal irrigating systems.

## Methods

The study was approved by the College of Dentistry Research Centre (CDRC), King Saud University, Riyadh, Saudi Arabia. Fifty extracted human single-rooted teeth were used in this study. The inclusion criteria were single-rooted mandibular premolar teeth with one root canal and one apical foramen. Teeth were then x-rayed buccolingually and mesiodistally to assess the patency of the root canal. The following were criteria for inclusion: non-carious teeth, completely formed apices, non-calcified canals, and canal curvature less than 20 degrees, which were determined according to Schneider’s method [11]. The root end was inspected under magnification (X 20) to verify closed apices and the absence of root resorption or visible cracks.

The root surfaces were cleaned of debris using a sharp scalpel. The teeth were radiographed from bucco-lingual and mesio-distal views to ensure they had single canals and orifices. The teeth were then stored in saline. A standard endodontic access cavity preparation was made into a pulp chamber using a carbide bur. The occlusal surfaces of the crowns were then flattened to achieve a standardized reference point to determine the working length. The working length (WL) was determined by inserting a K-file size #15, which was observed to extend beyond the apical foramen and then subtracting 1 mm from the length of the file.

The teeth were then randomly divided into three experimental groups according to the irrigation technique used and one control group for the cleanliness evaluation. Random allocation was done using lottery method.

• Group 1 (n = 15), irrigation was performed using the Endovac irrigation system (Discus Dental, Culver City, CA).

• Group 2 (n = 15), irrigation was performed using a 30-gauge, tip-vented irrigation needle (NaviTips, Ultradent, South Jordan, UT).

• Group 3 (n = 15), irrigation was performed using a 30-gauge, side-vented irrigation needle (Maxi-i-probe, Dentsply, Rinn, Elgin, IL).

• The control group (n = 5) received instrumentation, with no irrigation serving as a control for cleaning efficiency.

The outer surface of the roots was then coated with two layers of nail polish (except the apical 2 mm of the root) to control the transport of the irrigation solution via any lateral canals. The teeth were then mounted in a cylinder-shaped stone with a 10-mm diameter. The top end of the stone was levelled with the cemento-dentinal junction (CDJ), while the bottom end fell 2 mm short of the apical tip of the root. The whole assembly was then seated on a copper mould with the same diameter as the stone, where the exposed apical part of the root was contained within a 3-mm hole to collect the extruded irrigation solution. The interface between the stone and the copper mould was sealed with wax at the sides and with cellophane at the base, exposing only the hole that collected the extruded irrigation solution. All of the procedures were performed by one operator.

Instrumentation in all of the experimental and control groups was initiated using size 4, 3 and 2 Gates Glidden drills (Dentsply Maillefer, Ballaigues, Switzerland) for coronal enlargement. Hand instrumentation using a size #15 K-file was performed to the full WL. ProFile® rotary NiTi files (Maillefer Dentsply, Ballaigues, Switzerland) were used with a crown-down instrumentation technique. A controlled slow-speed, high-torque motor with a continuous speed of 300 rpm was used for the rotary files. ProFile® rotary files with a 0.06 taper were used starting with size 40, 35, 30, and 25, reaching an apical preparation of size #40. Lastly, hand instrumentation with size a #40 K-file was performed. The canal patency maintained using a size #10 K-file that was longer than 1 mm beyond the WL, was used after a profile size 25/0.06.

Irrigation in group 1 was performed using the EndoVac irrigation system, and the technique used was according to the manufacturer’s instructions. Irrigation was started using the Master Delivery Tip (MDT) at the access site and dispensing 1 ml of NaOCl each time after using a size 4, 3, and 2 Gates Glidden. The macrocannula was then used and placed inside the canal to approximately 3–4 mm from the WL to dispense the same amount (1 ml) of NaOCl after each endodontic file. At the same time, the master delivery tip was placed at the access site to continue irrigating. Again, 1 ml of NaOCl was delivered after each endodontic file. Each canal was cleaned and irrigated simultaneously for 30 seconds. Then, the master delivery tip was removed quickly approximately 1 second after removing the macrocannula to leave the canals charged with fresh irrigant. Lastly, the MDT was returned to continue irrigating at the access site, while placing the microcannula inside the canal 2 mm from the WL for 6 seconds. The microcannula was then moved down to the WL and held in position for 6 seconds. This process was repeated for a total of 3 cycles per canal, delivering 1 ml of NaOCl each time.

Irrigation in groups 2 and 3 was performed using tip-vented and side-vented irrigation needles, respectively. Both types of needles were first adapted for a disposable plastic syringe. Irrigation was started after using the Gates Glidden and then after all of the endodontic files by dispensing 1 ml of NaOCl solution each time. The needle was inserted as far as possible into the root canal with an up and down movement, up to 2 mm from the WL, without binding to the canal walls. The time of irrigation was constant for all of the canals, 30 seconds for each 1 ml dispensed.

All the experimental teeth received the same amount of 5.25% NaOCl irrigation with a total amount of 12 ml of NaOCl.

### Apical extrusion evaluation

The copper moulds were weighed before seating the moulded teeth on them using an electronic balance (Precisa 180A - Swiss made) to the fourth decimal. This value was then compared to the post-instrumentation weight of the moulds after removing the moulded teeth. The amount of extruded irrigating solution was then measured by subtracting the post-instrumentation weight from the pre-instrumentation weight.

### Cleanliness evaluation

The method for the cleanliness evaluation was a modified version of Al-Hadlaq et al. [18]. The teeth were removed from the stones and sectioned into two halves using a carborundum disk to create the longitudinal grooves on the buccal and lingual surfaces without entering the canals. The teeth were then split using a chisel and mallet. The most visible part of the canal was taken and divided into three main parts (coronal, middle, and apical) by creating three horizontal grooves and using a tapered carbide bur perpendicular to the canal. The samples were air-dried, sputter-coated with gold using a fine-coat ion sputter JFC-1100 (Fine coat ion sputter JFC-1100, JEOL Ltd., Tokyo, Japan), and then evaluated using Scanning Electron Microscope (SEM) (Jeol JSM-6360 LV, JEOL Ltd.). These three main parts were magnified up to X 20 magnification using SEM. Four random areas of each third were selected and magnified up to X 200 and then averaged to observe the debris layer removal from the canal walls. The captured images were analysed using ImageJ software (ImageJ 1.47 V, National Institute of Health, USA). The percentage of debris on the entire surface area was measured using the software to analyze the particles.

### Statistical analysis

Statistical analysis was performed using the SPSS software package (Version 16, SPSS Inc., Chicago, Illinois, USA). The descriptive analysis for the sample, mean values, range and standard deviation were calculated. An apical extrusion evaluation of the three different irrigating techniques was performed using the t-test and a one-way ANOVA. A cleanliness evaluation of the three different irrigating techniques was compared using a one-way ANOVA. A post-hoc Tukey analysis and repeated measure test were used for multiple comparisons. The level of statistical significance was set at P < 0.05.

## Results

### Extruded irrigating solution

**Figure 1:**
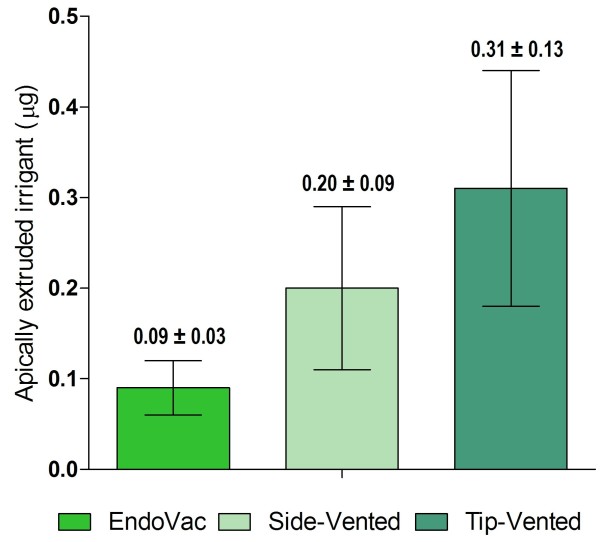
Weight (Mean ± SD) for the extruded irrigant for the EndoVac, side-vented, and tip-vented irrigation systems.

All forty-five teeth in the three experimental groups were included in the extrusion analysis to calculate the mean weight difference for the extruded irrigant. The mean weight of the extruded irrigant for the three experimental groups was highest for the tip-vented group (0.31 ± 0.13) followed by the side-vented group (0.20 ± 0.09) and then the EndoVac group (0.09 ± 0.03). Statistical analysis showed a significantly higher extrusion for the tip-vented and side-vented irrigation systems compared to the EndoVac (P <0.01). The side-vented group also had a significantly higher extrusion compared to the tip-vented group (P < 0.01) (Figure 1).

### Debris removal

**Figure 2:**
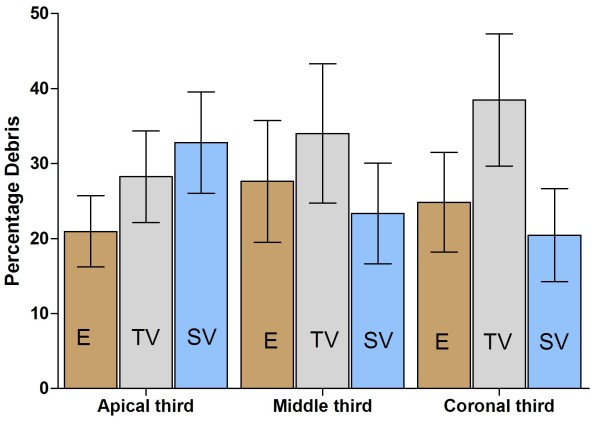
The percentage of debris (mean ± SEM) in the apical, middle and coronal thirds of the root canal using the three systems (E-EndoVac, TV-Tip Vented, SV- Side Vented).

**Figure 3:**
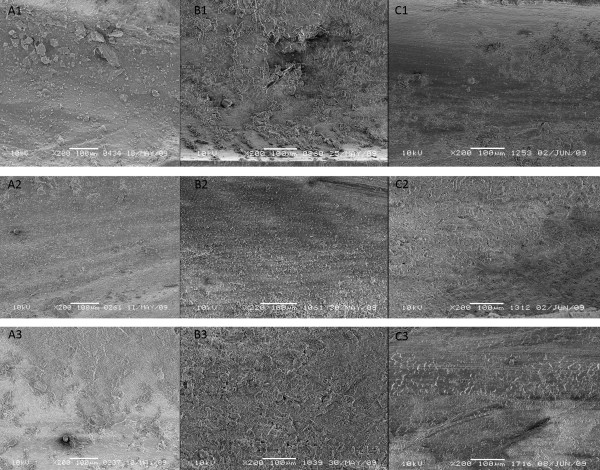
Scanning electron microscope images (x200) of the apical, middle and coronal thirds of the root canal using the three systems used (A1, A2, A3 - EndoVac; B1, B2, B3 - Tip Vented; C1, C2, C3 - Side Vented).

All fifty teeth in the three experimental groups and the control group were included in the cleaning analysis. The amount of the remaining debris in the coronal, middle and apical thirds was assessed, and the percentage of debris was calculated. The results are shown in Table 1 and Figures 2 and 3. The EndoVac system had minimal debris in the apical third followed by the coronal and middle thirds. The tip-vented needle irrigation system also had the least debris in the apical third (28.23%), followed by the middle third (34.01%), and then the coronal third (38.47%). For the side-vented needle irrigation, the mean debris score was the least for the coronal third (20.45%), followed by the middle third (23.35%), and then the apical third (32.79%).

**Table 1:** Percentage of debris in the apical, middle and coronal thirds of the root canal using the three systems

**Region of the root Canal**	**EndoVAC**	**Tip-vented**	**Side-vented**	**Control**
	**Mean ± SEM**	**Mean ± SEM**	**Mean ± SEM**	**Mean ± SEM**
Apical third	20.95 ± 4.75	28.23 ± 6.12	32.79 ± 6.76	96.18 ± 0.67
Middle third	27.62 ± 8.12	34.01 ± 8.78	23.35 ± 6.72	93.62 ± 0.93
Coronal third	24.83 ± 6.65	38.47 ± 8.80	20.45 ± 6.20	92.81 ± 0.51

## Discussion

This *in vitro* study was conducted to evaluate the effectiveness of the root canal debridement after irrigation with an EndoVac compared to the tip-vented and side-vented needle irrigation systems. The second objective of the study was to evaluate the extrusion of irrigants to the apical area of the root.

In the present study, the maximum debris removal in the apical third was achieved with the EndoVac followed by the tip-vented and side-vented irrigation systems. This observation is in agreement with previous studies, which showed that the EndoVac system is safer and more effective for cleaning the root canal [1,12,14,19,20].

The intracanal aspiration technique was developed to avoid the effects of irrigant extrusion [14]. EndoVac is an irrigation system that has been reported to have the ability to prevent the extrusion of irrigation solution and clean the entire root canal. The irrigation needle is inserted to working length and connected to the EndoVac suction device, which creates a negative pressure to aspirate the irrigation solution at the apex. This suction creates a steady flow of irrigation solution through the entire root canal, which allows the irrigant to debride and disinfect especially in last millimetre of the root canal without extrusion. A study by Fukumoto et al. [19] found that the negative pressure system had less extrusion of irrigant than needle irrigation (positive pressure) when both were placed 2 mm from the working length. Brunson et al. [20] studied the effect of apical preparation size and preparation taper on the volume of irrigant delivered to the working length of a root canal preparation. A statistically significant increase in irrigant volume was observed when apical preparation size increased from ISO #35 to ISO #45. They concluded that the root canal preparation to ISO #40 with a 0.04 taper seems to maintain a good balance of tooth structure preservation and adequate volume of irrigation at the apical third when using the apical negative pressure irrigation system.

The EndoVac system has been shown to provide better cleaning and disinfection of the root canal, especially in its apical third where the most debris is found [21,22]. EndoVac works by applying apical negative pressure at full working length to overcome the dangers of pushing irrigants outside of the root canal system. EndoVac is also expected to dry the canal by vacuum rather than the usual method of pressurised air. Parente et al. [23] compared efficiency of debris and smear layer removal in an open and closed root canal system using manual and EndoVac irrigation system. They concluded that the apical negative pressure irrigation is an effective method to overcome the fluid dynamics challenges inherent in closed canal systems.

The apical suction effect of the endodontic irrigants down and along the walls of the root canal creates a rapid turbulent cascade effect as the irrigants are forced to flow between the canal walls and the external surface of the microcannula. This turbulent action creates a current force, while the position of the microholes directs the fast-flowing stream of irrigant as close as 0.2 mm from the full working length before reversing the irrigant direction up to the microcannula [1,13]. Malentacca et al. [24] studied the efficacy and safety of an ultrasonic needle operating by aspirating sodium hypochlorite from the canal and to compare this with other systems, namely passive ultrasonic irrigation, EndoVac, and the ultrasonic needle operating in injection mode. Even though EndoVac extruded the least irrigant, the ultrasound needle working by aspirating sodium hypochlorite at a distance from the apex of more than 2 mm was found to be the best in terms of efficiency and safety.

Several methods have been used to evaluate the cleaning effectiveness of the irrigation, but some of these methods have many limitations. Analysing the hydroxyproline content of the irrigant yields excellent quantification but does not permit a visual inspection of the canal or the condition of the walls and tubules [25]. Evaluating the irrigation effectiveness by flushing the bead form gel has the disadvantage of the bead particles settling in the apical region, and gravity preventing the canal from filling with a homogenous fluid [26]. The radiographic images of radiopaque contrast medium exposed as the material clears from the canal are difficult to interpret [27]. Hence, in the present study, a scanning electron microscope was used to evaluate the presence of debris in the wall of the root canal after using different irrigation systems. The debris was evaluated using ImageJ software, which gave accurate results. The software is extensively used in medical imaging and analysis because of its functions such as edge detection, particle analyses and other sophisticated operations in all image formats and the capability of converting to quantitative measures [28]. In our study, the minimal apical extrusion of the irrigant solution using the EndoVac method is in agreement with earlier studies [3,17,19]. Mitchell et al. [29] found that the frequency of apical extrusion of the irrigant was dependent on the type of root canal irrigation system and the size of the apical preparation. The syringe and slotted-needle irrigation resulted in the greatest extent of extrusion.

The cleaning efficiency of the three irrigation systems was assessed by debris removal using a SEM evaluation of the coronal, middle and apical thirds of the canal. The EndoVac and tip-vented systems had the maximum debris removal at the apical third of the root canal [1,13,30]. Nielsen and Craig Baumgartner [14] compared the cleanliness efficacy between the EndoVac irrigation system and a 30-gauge ProRinse irrigation needle by determining the amount of remaining debris. At 1 mm from the working length, significantly better debridement of the root canal was achieved by the EndoVac system compared to needle irrigation, but no significant difference in debridement was found between the groups at 3 mm from the working length. The results of this study also showed no significant difference in the cleaning effectiveness between the three irrigation methods, which is in agreement with other studies [1,19,31]. The depth and volume of irrigation are important factors in removing debris and bacteria than the method used [8,20]. Howard et al. [7] compared the debris removal with EndoVac, PiezoFlow and needle irrigation. They found that with similar volumes of irrigant, all the three systems significantly improved canal and isthmus cleanliness.

The side-vented group in our study produced cleaner canals at the coronal and middles thirds compared to the tip-vented group, which is in agreement with other studies that showed the perforated endodontic irrigation needles had a greater distribution of irrigating solution and cleaner canals than a conventional irrigation needle [32-34]. Susin et al. [35] studied the canal and isthmus debris debridement efficacies of the manual dynamic irrigation and apical negative pressure techniques in the mesial root of mandibular first molars with narrow isthmi, using a closed canal design. Both techniques were not effective in the complete removal of debris from the isthmus regions. The EndoVac system removed considerably more debris from narrow isthmi in mandibular mesial roots. The efficacy of EndoVac in removal of smear layer and debris in closed canal is more than the manual dynamic agitation method [23].

This experiment was an *in vitro* study using extracted teeth. Thus, the extrusion results of this study may be different if they were applied to living teeth in the presence of periapical tissue that may resist the apical extrusion of irrigants and debris in vivo [36]. Moreover, mature teeth with closed apices were used in this study; therefore, the results should not be generalised to immature teeth with open apices.

## Conclusions

Within the limitations of the study, the EndoVac irrigation system was found to have extruded significantly less irrigant solution than the needle irrigation systems. The Endovac system was more efficient in removing debris from the apical third of the root canal compared to the tip-vented and side-vented irrigating systems.

## Competing interests

The authors declare that they have no competing interests.

## Authors’ contributions

AK and TDA carried out the study, endodontic procedures and evaluation of the results. SA was involved in the development of the concept, design of the study, revision of the manuscript and statistical analysis. All authors read and approved the final manuscript.

## Authors’ information

AK is an Associate Professor and TDA is a Lecturer in the Department of Restorative Dental Sciences, SA is a Professor in the Department of Periodontics and Community Dentistry, College of Dentistry, King Saud University, Riyadh, Kingdom of Saudi Arabia.

## Pre-publication history

The pre-publication history for this paper can be accessed here:

http://www.biomedcentral.com/1472-6831/14/12/prepub
